# Competing Frames in Global Health Governance: An Analysis of Stakeholder Influence on the Political Declaration on Non-communicable Diseases

**DOI:** 10.34172/ijhpm.2020.257

**Published:** 2021-01-16

**Authors:** Mao Suzuki, Douglas Webb, Roy Small

**Affiliations:** ^1^Department of Political Science and International Relations, University of Southern California, Los Angeles, CA, USA.; ^2^HIV, Health and Development Group, Bureau for Policy and Programme Support, United Nations Development Programme, New York City, NY, USA.

**Keywords:** Global Health Governance, Frames, Non-communicable Diseases, United Nations, Multi-Stakeholder Consultation

## Abstract

**Background:** Non-communicable diseases (NCDs) are increasingly recognized as a significant threat to health and development globally, and United Nations (UN) Member States adopted the Political Declaration of the Third High-level Meeting (HLM) on the prevention and control of NCDs in 2018. The negotiation process for the Declaration included consultations with Member States, intergovernmental organizations (IGOs), and non-state actors such as non-governmental organizations (NGOs) and the private sector. With NCD responses facing charges of inadequacy, it is important to scrutinize the governance process behind relevant high-level global decisions and commitments.

**Methods:** Through a review of 159 documents submitted by stakeholders during the negotiation process, we outline a typology of policy positions advocated by various stakeholders in the development of the Declaration. We document changes in text from the draft to the final version of the Declaration to analyse the extent to which various positions and their proponents were influential.

**Results:** NGOs and low- and middle-income countries (LMICs) generally pursued ‘stricter’ governance of NCD risk factors including stronger regulation of unhealthy products and improved management of conflicts of interest that arise when health-harming industries are involved in health policy-making. The private sector and high-income countries generally opposed greater restrictions on commercial factors. The pattern of changes between the draft and final Declaration indicate that advocated positions tended to be included in the Declaration if there was no clear opponent, whereas opposed positions were either not included or included with ambiguous language.

**Conclusion:** Many cost-effective policy options to address NCDs, such as taxation of health-harming products, were opposed by high-income countries and the private sector and not well-represented in the Declaration. To ensure robust political commitments and action on NCDs, multi-stakeholder governance for NCDs must consider imbalances in power and influence amongst constituents as well as biases and conflicts in positioning.

## Background

**Key Messages**

** Implications for policy makers**

- National and international policy-makers need to recognize that emphasis on inclusion of a range of stakeholders – particularly alcohol, food and beverage industries – in United Nations (UN) consultation processes on non-communicable diseases (NCDs) undermines the development of robust political commitments on NCDs.
- There is a need to reconsider inclusion/exclusion criteria in consultation processes for global policy-making and governance on NCDs.
- More broadly, conflicts of interest must be identified, evaluated for irreconcilability and closely managed both at the national and international levels.

** Implications for the public**

Non-communicable diseases (NCDs) are increasingly recognized as a significant threat to health and development globally, but governance of these diseases and their risk factors has been characterized by slow progress. This paper shows that the current form of multi-stakeholder consultation is contributing to the insufficient governance, as it incorporates the private sector, including the alcohol, food and beverage industries, and thereby delays cost-effective interventions, such as taxation of unhealthy commodities. This research can help policy-makers and public health advocates to consider new ways of negotiating responses to NCDs. A better policy-making environment will ultimately improve the health of people around the world.

On September 28, 2018, the United Nations (UN) General Assembly hosted the Third High-level Meeting (HLM) on the prevention and control of non-communicable diseases (NCDs), following HLMs on NCDs in 2011 and 2014. The 2018 HLM was attended by 23 heads-of-government and 55 ministers of health as well as intergovernmental organizations (IGOs), non-governmental organizations (NGOs), academia and the private sector. The HLM resulted in a new Political Declaration (the Declaration).^1^ Despite the series of high-level gatherings and declarations, NCD responses have been inadequate; characterized by slow progress in the development and sustainable financing of national response frameworks, and inadequate normative policy progress at the global and regional levels.^2-5^ This paper considers a potential reason for inadequate NCD responses by assessing how the global policy process for NCDs is influenced by multi-stakeholder consultation.

 The elevation of ‘intersectoral’ governance, the ‘whole-of-society approach,’ and public private partnerships (PPPs) in the era of the UN sustainable development goals has provided a welcoming environment for non-state actors, including the alcohol, food and beverage industries, to participate in multi-stakeholder consultations for NCD policy-making.^6,7^ However, as noted in an assessment of the negotiation of the ‘Montevideo Roadmap 2018-2030’ on NCDs in 2017, open and broad consultations that include commercial entities carry risks, such as softening the language towards health-harming commodities and reducing the ambitions of global policy.^8^ Some observers criticized the inclusion of commercial entities in the 2011 HLM as ‘representatives’ of civil society, and called for a code of conduct to manage conflicts of interest.^9-11^

 With concepts such as ‘commercial determinants’ and ‘industrial epidemic’ gaining currency, attention to industry engagement in the governance of NCDs has increased.^12-14^ Yet a large part of the growing literature describing industry interference is focused on the national or regional level,^15-17^ and thus there is a need to assess the role of industry at the global level.^7^ In this context, the 2018 HLM provides a major opportunity to examine how NCD policy-making at the global level is negotiated and influenced by various types of stakeholders.

 The consultative process of developing the Declaration enables us to examine the influence of different types of stakeholders through publicly available information. Ahead of Member State negotiations, a draft Declaration (zero draft) was prepared by co-facilitators based on a ‘pre-zero’ draft produced by the World Health Organization (WHO).^18^ Non-state actors commented on the draft during an interactive hearing on July 5.^19^ During the HLM, Member States and non-state actors provided statements, many referencing the final Declaration.^20^ Based on the documents submitted to the interactive hearing and the HLM, we conduct a frame analysis and identify main messages and positions of different stakeholders in relation to addressing NCDs.

 Framing is widely recognized as an important strategy to influence policy debates,^21-23^ but the concepts of ‘framing’ and ‘frame’ are used in different ways in various academic disciplines. Because frames serve multiple purposes, scholars have attempted to classify them at various ‘levels of abstraction.’^23^ Koon et al mention that frames can be classified based on whether they define, diagnose, judge or prescribe; they therefore recognize the functions of framing from a very abstract level (ie, understanding an issue in a certain way) to a relatively concrete level (ie, providing certain policy recommendations).^23^ Within this broad spectrum of the concept of framing, we focus on concrete policy positions as a reflection of certain cognitive assumptions that different types of stakeholders promoted during the consultation process. By combining our analysis with the existing studies on the framing of NCDs, we argue that understanding the dissonance between the disease burden and global response requires recognizing competing policy positions and the power imbalance that the current consultations among multiple stakeholders uphold.

 Market-oriented policies encourage unfettered trade in products that are or may be harmful to public health, specifically tobacco, alcohol and unhealthy foods and beverages, as well as weak legislative and oversight mechanisms to address these risk factors.^24^ Free-market capitalism maintains the primacy of the individual consumer over the responsibility of state or economic actors to incentivize healthier consumption through regulation. As such, public health detriments are viewed as failures of individual choice-making rather than as dereliction of governmental duty to ensure health-enabling environments and the right to health by addressing externality-induced market failure.^25^ This neoliberal position is generally reinforced by institutions benefiting from market-oriented policies and challenged by health and development advocates concerned about the social, economic and environmental harms related to NCDs. For instance, in their assessment of why incoherence between NCD policy and trade policy endures in Australia, Battams and Townsend found that policy actors in each area were using fundamentally different frames, which was perceived to impede effective coordination between the two sectors.^26^ Given that policy actors in the trade sector often view individuals as fundamentally responsible for their own health, a trade policy which strongly regulates importation of health-harming commodities is unlikely. Weishaar and colleagues also identified two competing frames in the media coverage of NCDs; corporations attempt to promote the “market justice frame” (which essentially reflects free-market capitalism and deregulation), while public health advocates endorse the “social justice frame” which emphasizes the social and political determinants of health and the need for regulations.^27^

 This paper assesses if there is a similar competition among neoliberal and other cognitive assumptions (represented as competing policy positions) in the consultative process at the global level and, if there is such a competition, how it affects the policy outcome. Inevitably, patterns of power distribution manifest in how ideas are communicated, represented and reflected in global decisions. In relation to the Declaration, the objectives of this paper are (1) to identify the main policy positions that different stakeholders presented regarding the prevention and control of NCDs as inputs into the overall drafting and endorsement process, and (2) to illustrate how influential these policy positions were during its formulation.

 Though our main concern in this paper is the potential influence of the private sector, we fully acknowledge that the final text of the Declaration was negotiated and agreed upon by Member States. As stated in resolution A/RES/72/274, which set the scope, modalities, format and organization of the third HLM, the meeting was to “approve a concise and action-oriented outcome document which builds on the opportunities and challenges in the implementation of previous commitments, *agreed in advance by consensus through intergovernmental negotiations*” (emphasis added).^28^ This means that even though non-state actors could voice their opinions in the interactive hearing and at the HLM, these actors ultimately cannot officially authorize over the decision about the Declaration. Indeed, actors such as NGOs and the private sector did not have an official route to communicate their opinions after the interactive hearing, and the final draft of the Declaration was produced at the end of “active and constructive engagement” of the Member States and “extensive consultations with delegations.”^29^ That said, if invited by governments, NGOs and private-sector actors can be part of country delegations which would enable them to advocate for their positions during intergovernmental negotiations.

 The final draft of the Declaration was submitted by the co-facilitators of the intergovernmental negotiations to the President of the General Assembly on September 18. The draft was put under silence procedure until the following day, during which delegates of the Member States could break consensus on the document and bring it back into negotiation. Non-state actors did not have the authority to break this silence. The Member States remained silent during the period, and the final draft became the official Declaration on September 19.

 Considering this procedure and the limited official role non-state actors played, we do not claim that the submissions made to the interactive hearing are the only inputs that affected the contents and the wording of the final Declaration. Our aim is to assess whether there is any corresponding pattern between the changes in text of the Declaration and the sources of the policy positions presented during the consultation process. And, if the positions of certain stakeholders are overly reflected in the evolution of the Declaration, we consider it reasonable to infer that the stakeholders influenced global policy-making, even if they are non-state actors who had no ‘official’ authority to draft the Declaration.

## Methods

 This paper reviews 159 documents in total. Most of the statements were from NGOs and academic institutions (99 statements, 62% of submissions), followed by Member States (37 statements), IGOs (14 statements) and the private sector (9 statements). All statements by Member States were provided during the HLM, while more than half of the submissions from NGOs and academic institutions were shared in the interactive hearing. Contributing Member States span country-income levels, yet low- and middle-income countries (LMICs) account for 65%. Participants from the private sector include the following industries: food and beverage (4 statements), alcohol (2 statements), pharmaceutical (2 statements), and consulting (1 statement).

 For the interactive hearing, convened by the President of the UN General Assembly with the WHO, 72 documents commenting on the draft Declaration were submitted to the WHO by NGOs and academic institutions (61 submissions), the private sector (6), and IGOs (5). These documents are publicly available online.^19^ Another 98 statements were submitted to the HLM commenting on the final version of the Declaration, by Member States (46 statements), NGOs and academic institutions (38), IGOs (10), the private sector (3), and multi-stakeholder entities (2). These documents are also publicly available.^20^ Among the 46 statements submitted by Member States during the HLM, the statements in French (Burkina Faso, Cameroon, Cote d’Ivoire, Central African Republic, Madagascar, Mauritania, and Senegal) are omitted due to limits in translation. Statements from GAVI and the Coalition for Access to NCD Medicines and Products were not analysed because these are only two submissions from niche multi-stakeholder coalitions which do not fit one of our 4 larger categories. The analysis also excludes 4 submissions without specific reflection on the final or draft Declaration: submissions by Paiman Alumni Trust (interactive hearing), ABRALE Red Alianza Latina (interactive hearing), Belgium (HLM), and Panama (HLM).

 To determine the positions of different stakeholders (Member States, IGOs, NGOs, academia and the private sector), all data sources were coded to summarize main points and identify core framing elements, with the positions inductively generated by reading the texts. For instance, the sentence “[I]n order to boost financing for NCDs prevention and control, the importance of domestic resource mobilization cannot be overstated” (International Organisation of Good Templars) is identified as a message of “domestic funding is important.” Similarly, “[I]n this context, alcohol taxation holds massive potential for beating NCDs” is coded as “support for tax on alcohol.” The positions were then reviewed to eliminate substantive duplicates. Positions were merged where they had substantively similar messages. For example, support for tobacco tax, alcohol tax, and regulating advertisements of health-harming commodities were merged into a single position as “support regulation of harmful products.” The coding produced 56 distinct positions in total. Some organizations submitted statements for both the interactive hearing and the HLM, and we treat these statements separately. Therefore, the positions could be counted twice when an organization endorsed the positions in both its interactive hearing and HLM statements.

 Though some stakeholders use “multi-sectoral,” “multi-stakeholder,” “whole-of-society,” and “health-in-all” approaches interchangeably, we distinguish them. Here, “whole-of-government,” “health-in-all,” and “cross-ministry” indicate collaboration among governmental (public) entities, while “whole-of-society” and “multi-stakeholder” indicate cooperation among state and non-state actors including civil society and the private sector. PPPs is a subcategory of whole-of-society. This paper calls the former “support for ‘whole-of-government’ and ‘health-in-all policies’ approaches” and the latter “support for whole-of-society approach, including the use of PPPs.” An important conceptual distinction between these two approaches was elaborated elsewhere.^30^

 To assess the extent to which each stakeholder potentially influenced the Declaration, changes between the draft and final versions of the Declaration are assessed in relation to the stated positions of stakeholders during the consultative process. In comparing the draft and final versions of Declaration, this paper identifies: (1) themes not in the draft but included in the final Declaration (‘clear additions’); (2) themes added to the final Declaration but lacking clarity (‘equivocal language’); and (3) themes not emphasized in either version despite advocacy (‘omissions’).

## Results

###  Policy Positions of Stakeholders

**Figure 1:**
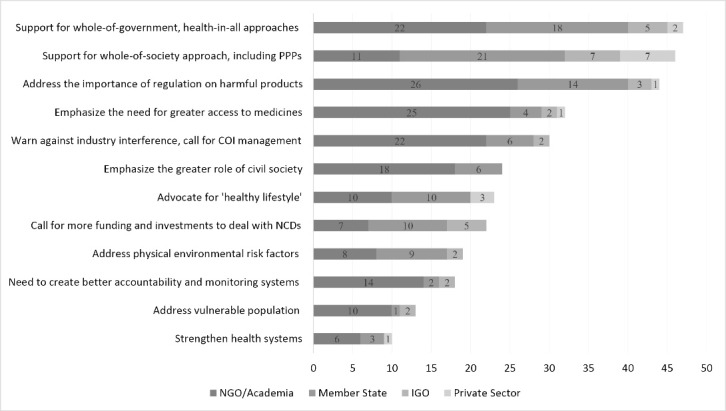
Main Feedback Themes From Stakeholders. Abbreviations: PPPs, public private partnerships; COI, conflicts of interest; NCDs, non-communicable diseases; IGO, intergovernmental organization; NGO, non-governmental organization.

 Main positions were included in Figure 1 if mentioned in at least 10 submissions. The most frequent position is support for whole-of-government and health-in-all approaches (47 submissions, 30% of all submissions). This theme is endorsed across stakeholders, showing consensus on a comprehensive NCD response in which various governmental bodies cooperate. Following this, support for a whole-of-society approach specifically inclusive of PPPs is mentioned in 46 statements (29%), also from all stakeholders but mainly by the private sector, IGOs and Member States. The importance of regulations on harmful products (44 statements; 28%) is strongly represented by NGOs and academic institutions, with some but lesser endorsement from Member States, IGOs and the private sector. This theme includes support for a ban or other restriction on advertisement and sales, rules on labelling, and taxes on tobacco, alcohol, and/or unhealthy foods. Submissions endorsing at least one of these measures are considered supportive of regulations on harmful products.

 The need for greater access to medicines, vaccines, palliative care and other facilities is addressed in 32 statements (20%). Five statements mention TRIPS (Trade-Related Aspects of Intellectual Property Rights) flexibility to improve access, and 6 statements suggest creating alternative pharmaceutical research and development (R&D) mechanisms to delink prices and R&D in the costs of medicines. The importance of managing conflicts of interest and caution against industry interference is in 30 statements (19%). Twenty-four statements (15%), mostly from NGOs and academic institutions, call for a greater role for civil society, people living with NCDs and their families, communities, indigenous people, and youth.

 ‘Healthy lifestyle’ is promoted in 23 statements (14%), endorsing changing individual behaviours, awareness raising, public education, and physical activity. This position is mainly presented by Member States, NGOs and the private sector. Twenty-two statements (14%) call for greater funding, domestic and/or international, to address NCDs. This is mostly supported by Member States, especially LMICs.

 The importance of addressing environmental risk factors such as air pollution and climate change (19 submissions, 12%), and the need to create better accountability and monitoring systems (18 submissions, 11%), are also mentioned. The latter is predominantly raised by NGOs and academic institutions, while the former attracts broad consensus among Member States, IGOs, NGOs and academic institutions. Thirteen statements (8%) highlight concerns for vulnerable populations such as women, girls, indigenous people, children, people with disabilities and people in humanitarian crises, and 10 statements (6%) emphasize the importance of stronger health systems.

###  Private Sector

**Figure 2:**
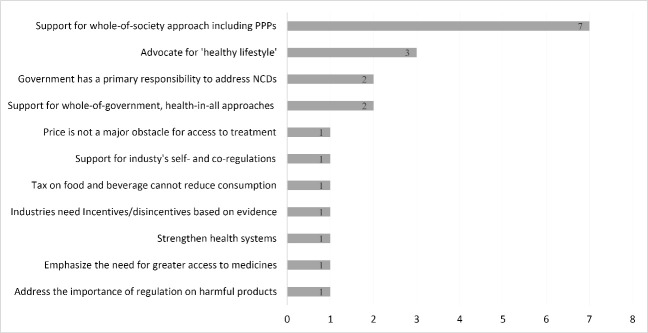
Private Sector Messaging. Abbreviations: PPPs, public private partnerships; NCDs, non-communicable diseases.

 The main private sector message is that multi-stakeholder partnerships are “necessary,” “essential,” or “indispensable” to address NCDs and that the private sector has an important role to play. Seven out of 9 private sector submissions (78%) support the greater use of PPPs. Three statements from the private sector (33%) point out the importance of ‘healthy lifestyle;’ this is consistent with the neoliberal model discussed earlier. Two entities support collaborations among governmental bodies beyond the health sector. Two submissions from the pharmaceutical industry said that government has the primary responsibility to address NCDs and achieve universal health coverage. The private sector raised additional ideas (Figure 2), reflecting somewhat diverging interests of different industries. The Coalition for Access, mainly composed of pharmaceutical companies, supports greater access to medicines as well as taxation of sugar-sweetened beverages (SSBs). Another pharmaceutical-related association, the International Federation of Pharmaceutical Manufacturers and Associations, explicitly states that price was not a major obstacle for access to insulin. The Alliance of Food and Beverage Associations in Latin America argues against SSB taxation, claiming that taxes on foods and beverages are not effective in reducing consumption. These messages suggest that private sector entities have different policy preferences. Still, wide support for greater use of PPPs shows agreement within this constituency that the private sector should be included in NCD governance.

###  Intergovernmental Organizations 

**Figure 3:**
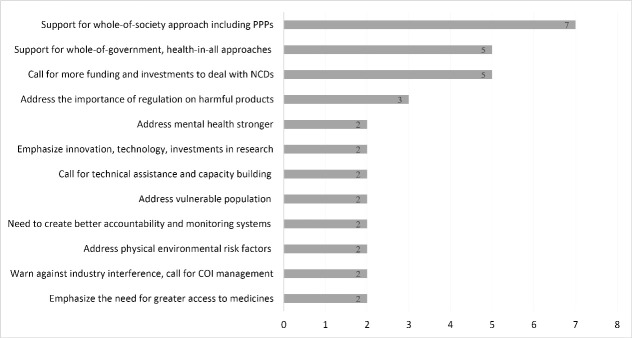
Intergovernmental Organization Messaging. Abbreviations: PPPs, public private partnerships; NCDs, non-communicable diseases; COI, conflicts of interest.

Figure 3 shows themes discussed in at least 2 statements from IGOs, ie, organizations of sovereign states, here: the Food and Agriculture Organization of the UN, International Atomic Energy Agency, Pacific Community, South Center, United Nations Population Fund, United Nations Office for Project Services, WHO, the President of the UN General Assembly, and the Deputy Secretary-General of the UN. Because each IGO statement represents its area of expertise or the position of affiliated Member States, messages from this constituency are less coherent than those of the private sector. Yet, support for a whole-of-society approach inclusive of PPPs (50% of IGO submissions), as well as whole-of-government and health-in-all policies approaches (36% of IGO submissions), are broadly endorsed by the IGOs.

 The IGOs covered several themes. The United Nations Population Fund emphasizes gender and the connection between NCDs and reproductive health, while the Food and Agriculture Organization addresses the importance of food systems and multi-stakeholder approaches. The International Atomic Energy Agency emphasizes its relevance to health through science, technology, and innovation. The UN Office for Project Services endorses a whole-of-society approach and greater use of PPPs. In contrast, the South Centre reiterates the voice of developing countries which are disproportionately affected by NCDs, the importance of regulating harmful products, and greater access to medicines. Its HLM statement mentions the need for greater funding and capacity building: “[T]his Declaration should be conducive to mobilize new financial resources and capacity building for developing countries that require support to enable them to meet the challenge posed by NCDs.” The Pacific Community highlights the situation in the Pacific, expressing strong support for the WHO ‘Best Buys’^31^ and referring to successful cases of taxation on tobacco, alcohol, and SSBs in its Member States.

###  Member States

**Figure 4:**
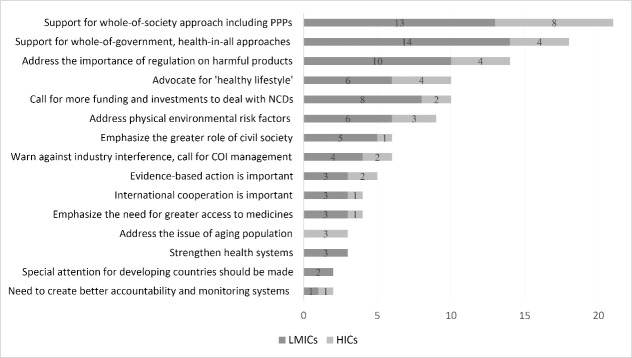
Member State Messaging. Abbreviations: PPPs, public private partnerships; NCDs, non-communicable diseases; COI, conflicts of interest; LMICs, low- and middle-income countries; HICs, high-income countries.

Figure 4 shows themes discussed in at least 2 statements from Member States. High-income countries (indicated by light grey bars) and LMICs (indicated by dark grey bars) stress different positions, but Member States generally agree on 2 themes: support for a whole-of-society approach (21 statements, 57% of Member State submissions) and support for whole-of-government and health-in-all policies (18 statements, 49%). It is worth mentioning, though, that a whole-of-society approach is supported by 62% of high-income countries while only 54% of the LMICs support the theme. In contrast, 58% of LMICs support a whole-of-government approach but only 30% of high-income countries support this theme. Therefore, there are seemingly diverging preferences between high-income countries and LMICs: high-income Member States are more supportive of the idea of a whole-of-society approach than a whole-of-government approach, whereas LMICs prefer the opposite.

 The major concern of LMICs is the need to regulate harmful products (10 out of 24 statements from LMICs), most notably through taxes on tobacco and alcohol. Other themes primarily raised by LMICs include the need for greater funding (8 LMIC statements), increased emphasis on the role of civil society (5 LMIC statements), and concerns regarding industry interference (4 LMIC statements).

 The notion of ‘healthy lifestyle’ is raised by 10 countries across all income levels, including high-income countries such as Canada and Japan. The importance of addressing environmental risk factors is also supported by both high-income countries and LMICs (9 statements from Member States). Small island developing states in the Pacific are the strongest advocates for this theme. Aging populations are addressed as an important aspect of NCDs by high-income countries (3 statements; Brunei, France and Japan).

###  NGOs/Academic Institutions

**Figure 5:**
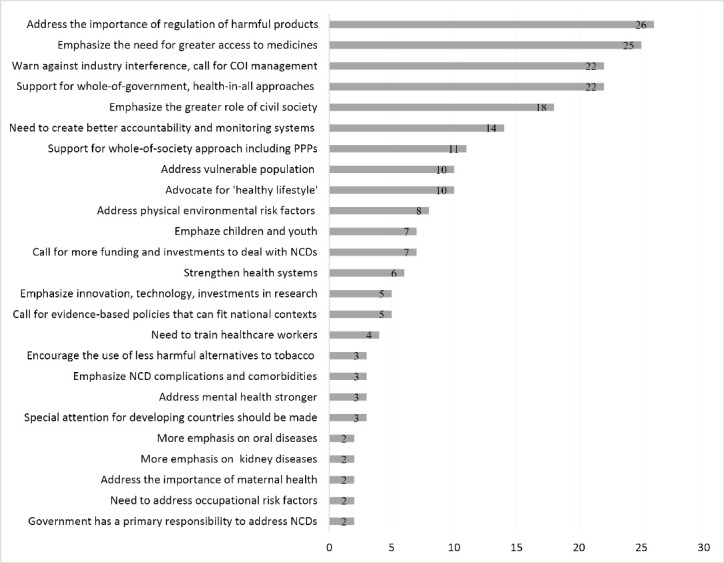
Non-governmental Organization Messaging. Abbreviations: COI, conflicts of interest; PPPs, public private partnerships; NCDs, non-communicable diseases.

Figure 5 shows themes discussed in at least 2 statements from NGOs and academic institutions. This constituency stresses regulation of health-harming products and greater access to medicines. Twenty-six statements (26% of NGO submissions) address regulations, with SSB taxation most popular (10 out of 26 statements support SSB taxation). Twenty-five statements advocate greater access to essential medicines.

 NGOs and academic institutions strongly support a whole-of-government approach (22 submissions) over a whole-of-society approach (11 submissions), while other stakeholders give these almost equal endorsements. There is broad agreement among NGOs that industry interference is problematic and that conflicts of interest must be managed (22 statements). NGOs and academic institutions also advocate for the greater role of civil society (18 statements), stronger accountability and monitoring systems (14 statements), and vulnerable populations (10 statements).

###  Evolution of the Political Declaration

####  Clear Additions 

#### Importance of Environmental Risk Factors (Para 31, 32)

 Language on environmental risk factors is expanded in the final Declaration compared to the draft. While the draft only contains the term “environmental determinants” once, the final Declaration adds two paragraphs on this. Paragraph 31 highlights indoor and outdoor air pollution as risk factors, and paragraph 32 further specifies “air, water and soil pollution, exposure to chemicals, climate change and extreme weather events, as well as the ways in which cities and human settlements are planned and developed, including sustainable transportation and urban safety, to promote physical activity, social integration and connectivity” as environmental determinants that Member States need to address.^1^ The importance of addressing environmental risk factors is the ninth most discussed position across all submissions (19 statements in total), and the major advocates are Member States (9 submissions) and NGOs (8 submissions). Small island developing states emphasize this theme with support from high-income countries such as Canada and Finland as well as IGOs. No entity expresses specific opposition.

#### Particular Concerns for Older Persons and the Aging Population (Para 13, 29)

 The draft does not mention the term “older persons” or “aging population,” while the final Declaration devotes two paragraphs to this theme. This theme is not widely advocated across submissions, with only 4 statements referencing it. Three out of 4 statements are from high-income countries (Brunei, France, and Japan), and Japan provides a lengthy comment on this theme.^20^

#### Vulnerable Populations (Para 14, 19, 28, 40)

 The health needs of specific populations are addressed more in the final Declaration than in the draft, with one paragraph added and two existing ones modified. The draft text did not mention “women,” “girls,” or “gender.” Paragraph 14, new, emphasizes a gender perspective and the impact of NCDs on women. While the operative paragraph (OP) 6 in the draft mentions the specific health needs of children only, paragraph 28 in the final Declaration, on the right to health, expands this to also include “women, older persons, persons with disabilities and others who are more vulnerable.” Paragraph 19, on country-led prioritization and implementation of interventions, added “a particular emphasis on the needs of those in vulnerable situations.” Paragraph 40 introduces the special concerns for people living with NCDs in humanitarian emergencies. The needs of vulnerable populations are supported in 13 submissions (the 11th most discussed theme), primarily from NGOs and academic institutions (10 statements). Indigenous peoples are specified in the submissions but omitted in the final Declaration.

#### Promotion of ‘Healthy Lifestyles’ (Para 33)

 An independent paragraph (33) in the final Declaration promotes healthy lifestyles and regular physical activity, while a similar sentence is provided as one of 3 options for OP12 in the draft. This theme appears in 23 stakeholder statements (the seventh most discussed theme). Member States and NGOs provided strongest support (10 statements each) followed by private sector (3 statements).

####  Additions with Equivocal Language

#### Access to Medicines and the TRIPS Flexibility (Para 36, 38)

 By adding two paragraphs on the need for greater access to essential medicines, the final Declaration emphasizes this more than the draft does. The draft mentions that “access to safe, affordable, effective and quality essential medicines and technologies” should be improved (OP13) and calls upon the private sector to “[C]ontribute to efforts to improve access to and affordability of medicines and technologies” (OP18-e). The final Declaration keeps these paragraphs and adds paragraph 36, which lists the Doha Declaration on the TRIPS Agreement as a tool to improve access, and paragraph 38, which promotes access to affordable medical products to reduce the risk of cancer.

 Access to medicines is the fourth most discussed theme (32 statements) and strongly addressed by NGOs, including Doctors without Borders and Knowledge Ecology International. Some Member States and IGOs also show support, indicating broad agreement on the importance of access to medicines; however, there is no consensus in the submissions on how to achieve this. While NGOs stressed “soaring drug prices” and “patents or other intellectual property rights” as major problems, the pharmaceutical industry rejects this view. Many NGOs express disappointment in what they view as weak language of the Declaration, especially because “the declaration does not acknowledge that prices for new drugs, vaccines, and diagnostics for NCDs are excessive or prohibitively high” (Knowledge Ecology International) and “the exploration of new approaches to financing R&D that would eliminate the conflict between innovation and access” (Union for Affordable Cancer Treatment) is missing.

#### Reducing “Harmful Use of Alcohol” and Eliminating Marketing to Minors (Para 44b, c)

 Despite extensive advocacy for regulatory instruments on health-harming products, strict regulations are not addressed in the Declaration; rather, 2 suggestions for *voluntary* alcohol control are incorporated. In paragraph 44, which addresses the role of the private sector in NCD prevention and control, 2 sub-paragraphs are introduced. Paragraph 44-b encourages alcohol producers to “contribute to reducing harmful use of alcohol in their core areas” and paragraph 44-c calls for elimination of “the marketing, advertising and sale of alcoholic products to minors.” These additions do not include binding commitments to regulations.

 Forty-four statements raise the importance of regulating products such as tobacco, alcohol, and unhealthy foods and beverages, one of the most common themes. Fourteen statements endorse taxation on alcohol and other WHO ‘best buys’ related to alcohol. Major supporters of alcohol regulations are Member States and NGOs (6 statements, respectively). An SSB tax generated even larger endorsement (18 statements, mostly from NGOs and academic institutions). The Declaration does not once mention “tax.” Paragraph 21 suggests “fiscal measures, as appropriate.”

#### Participation of All Relevant Stakeholders, Including the Private Sector (Para 15, 41, 46)

 Compared to the draft, the final Declaration shows stronger support for participation of “all stakeholders” – but with ambiguous language. In the final Declaration, immediately preceding the paragraphs on cooperation with non-state actors, a short new paragraph is inserted: “Pursue all necessary efforts to mobilize the full, active and responsible engagement and participation of all relevant stakeholders for the prevention and control of non-communicable diseases” (paragraph 41). Paragraph 15 in the final Declaration (on the primary role and responsibility of governments) is significantly expanded from the preambular paragraph 10 of the draft, underscoring the importance of whole-of-government and whole-of-society approaches. Similarly, paragraph 46 elaborates the importance of financing mechanisms and partnerships “including with the private sector,” while OP20 in the draft does not specify private sector partnerships.

 These changes relate to the two most discussed themes across submissions: support for a whole-of-government approach and support for a whole-of-society approach. The Declaration obscures the difference between these approaches by raising them consecutively and implying their interchangeability. This is against the view of many NGOs, which only consider a whole-of-society approach to include the private sector. Industries seem to interpret this ambiguity as a welcoming invitation:

*“The Declaration invites all actors, including the private sector, to step up their commitments and actions to find solutions to address the burden of NCDs, and we have pledged to do our part” *(International Food & Beverage Alliance).^20^

####  Omissions: Themes Not Reflected

#### Commercial Determinants of Health and the Management of Conflict of Interest

 Despite strong advocacy by NGOs, academic institutions and some Member States, better management of conflict of interest and caution against industry interference are not incorporated in the final Declaration. These points are together discussed in 30 statements (the fifth most supported theme). Eighteen statements (all but one from NGOs and academic institutions) explicitly emphasize the importance of managing conflicts of interest. The US Alcohol Policy Alliance expresses concern over the alcohol industry’s inclusion in the process, stating that “[T]hey should no more be considered as a part of the whole of society.” Some entities express disappointment with the outcome document: “The declaration encourages multi-sectoral partnerships, but the risks associated with conflicts of interest are mentioned only briefly” (Knowledge Ecology International). Similarly, although the need to address commercial determinants is explicitly supported in 17 statements, the Declaration does not mention “commercial determinants.” The American Heart Association admits “disappointment that the report did not call out the commercial determinants of health as a major obstacle to progress,” and mentions that “unhealthy commodity industries continue to use tactics that can undermine the role of governments to protect the public health of their citizens.”

#### More Funding and Resources

 Twenty-two statements, including 9 from LMICs, emphasize the need for greater financing (the eighth most supported theme). The Declaration mentions no concrete strategy, suggestion or commitment regarding financing. Paragraph 46 merely states the importance of “adequate, predictable and sustained resources,” as did OP20 of the draft. Mongolia, a Member State with limited resources, states that “[I]t is crucial to increase funding of not only local government, but also of international donor organizations.” The FDI World Dental Federation, an NGO, stated it was “disappointed in the 2018 Declaration which lacks any meaningful financing and investment commitments for NCDs.”

#### Accountability and Monitoring System 

 Eighteen statements emphasize the importance of better accountability and monitoring (the 10th most discussed theme). However, no concrete suggestion or mechanisms were added to the Declaration. Paragraph 45 in the final Declaration (and OP19 in the draft) address a transparency and accountability mechanism to monitor the control of NCDs, but do so generally. Given this outcome, the International Diabetes Federation mentions that it is “gravely concerned by the omission in the Political Declaration of the necessary monitoring mechanisms to track progress and make governments accountable.”

#### Call for a Greater Role of Civil Society 

 Call for a greater role of civil society is the sixth most discussed theme, and a major theme advocated by NGOs. Despite this, there are no significant changes between the final Declaration (paragraph 42) and draft (OP16) in how civil society is discussed.

## Discussion

 The analysis found that, generally, NGOs, academic institutions and LMICs pursue a ‘stricter’ form of governance of NCD risk factors, while the private sector and high-income countries oppose greater restrictions on corporate practice and promote a whole-of-society approach that includes collaboration with the private sector. This finding is consistent with the existing studies arguing that the private sector is promoting market-oriented policies backed by neoliberal assumptions, while public health advocates emphasize the social and commercial determinants of health and the role of public regulations.^26,27,32^ A comparison of draft and final versions of the Declaration revealed that these competing positions among – and within – stakeholders work to block the inclusion of cost-effective measures such as taxes on SSBs.

 As Table (indicated by light grey shade) shows, many themes mentioned in more than 30 submissions are highly contested among stakeholders, and the contested themes tended to be included in the Declaration using ambiguous language, if at all. The final Declaration adds language on the importance of access to medicines but does not specify how to achieve this. That is, the Declaration does not mention high prices as a problem, and suggestions by NGOs to delink the costs of R&D from pricing did not alter the text. Pharmaceutical companies clearly opposed the idea that price is a major obstacle for access to insulin. Regulation of harmful products is another area of dispute among stakeholders. While there was consensus from all constituencies including the alcohol industry to reduce the “harmful use of alcohol” and eliminate “the marketing, advertising and sale of alcoholic products to minors,” resulting in the inclusion of sub-paragraphs on these issues, there was strong opposition from the beverage industry to any SSB tax.

**Table:** Contestation, Advocacy level, and Reflection on the Declaration of the Major Themes

**Theme**	**Submission**	**Contested**	**Outcome**
Support for whole-of-government, health-in-all approaches	47	No	Ambiguous
Support for whole-of-society approach, including PPPs	46	Yes	Ambiguous
Address the importance of regulation on harmful products	44	Yes	Ambiguous
Emphasize the need for greater access to medicines	32	Yes	Ambiguous
Warn against industry interference, call for COI management	30	Yes	Ignored
Emphasize the greater role of civil society	24	No	Ignored
Advocate for 'healthy lifestyle'	23	No	Added
Call for more funding and investments to deal with NCDs	22	No	Ignored
Address physical environmental risk factors	19	No	Added
Need to create better accountability and monitoring systems	18	No	Ignored
Address vulnerable population	13	No	Added
Address the issue of aging population	4	No	Added

Abbreviations: PPPs, public private partnerships; COI, conflicts of interest; NCDs, non-communicable diseases.

 Participation of “all relevant stakeholders” is in the Declaration but with ambiguity as to who these relevant stakeholders are. The final Declaration also merely adds the term “conflict of interest” without specifying how to manage it. NGOs highlight the risks of engagement with the private sector, Big Alcohol and Food in particular, and make a strong call for management of conflict of interest. This position is promoted as an antithesis of “support for whole-of-society, including PPPs,” which is endorsed by other types of stakeholders. In the end, the latter position was added to the Declaration in a way that allows confusion with a more widely supported whole-of-government approach, and the strong warning against industry interference was ignored.

 In contrast, none of the themes that were clearly added to the final Declaration were contested among stakeholders (indicated by dark grey shade in Table). Inclusion of two clear, concrete paragraphs on environmental risk factors and specific language on vulnerable populations illustrate the importance of the absence of ‘opponents.’ Even though lack of contestation does not guarantee inclusion of a theme into the Declaration (as some themes such as the greater role of civil society were not included despite being unopposed), the pattern shown in Table implies that having no opponent is a more important criterion for inclusion than overall level of advocacy. Concern for vulnerable populations and the aging population are incorporated in the final Declaration *despite the relatively low level of support for these positions.*

 Another factor that possibly affected inclusion in or omission from the Declaration is the political cost for Member States, especially those with high incomes. Concern for older persons and the aging population is added in the final Declaration despite very low advocacy (only 4 statements), while the need for greater financing is excluded despite relatively strong advocacy (22 submissions). The inclusion of aging population would not pose much political cost while greater financing would inevitably present high political (and assumed financial) costs for high-income countries, because these countries may benefit from the inclusion of the former theme (they are experiencing population aging) but not from the latter (they may assume some obligation to fund NCD responses in LMICs through bilateral and multilateral channels). Indeed, all but two of the 10 Member States who support greater financing are LMICs whereas all 3 countries that express concern for their aging populations are high-income countries. This contrast (inclusion of aging population and omission of greater financing) hints at a power imbalance between high-income countries and LMICs.

 Whether they are based on neoliberal ideological assumptions or calculations of political costs, voices predominant in high-income countries and the private sector opposed calls for stricter policy options such as regulations of health-harming commodities that were advocated by NGOs and LMICs. Multi-stakeholder global health governance must acknowledge such power imbalances amongst constituents and recognize biases in their relative influence. Such biases can delay cost-effective response frameworks and robust political commitments to the extent that ‘consensus-based’ decision-making results in chronically insufficient progress.

 The fact that the current form of multi-stakeholder consultations is directing global NCD policy towards voluntary measures and PPPs instead of government regulation is alarming, especially considering the relative lack of evidence that industry self-regulation or PPPs are effective in reducing risk factors for NCDs.^13^ Moreover, a robust system for managing conflicts of interest has not been developed, despite the trend towards support for a whole-of-society approach. This is the worst possible combination (promotion of PPPs *without* the management of conflict of interest) from the perspective of many NGOs and public health advocates.

 An important question remains of who should be included in consultative processes for global policy-making. Is the inclusion of the private sector, alcohol, food and beverage companies in particular, still appropriate? There is a growing body of evidence that these industries are using tactics similar to those adopted by Big Tobacco to undermine public health efforts,^2,33-45^ some of which were seen in the analysis here, including questioning evidence of harm-reduction interventions. Yet, the WHO’s stance on the alcohol, food and beverage industries is weaker than its stance on the tobacco industry. Both the Global Strategy on Diet, Physical Activity and Health and the Global Strategy to Reduce the Harmful Use of Alcohol assume partnerships with these industries, which the FCTC prohibits.^46-48^ A recent WHO decision not to engage with the alcohol industry when developing alcohol policy or implementing public health measures should be welcomed,^49^ but what might be more challenging is building a firm stance against Big Food and Soda. A survey conducted by Collin and his co-authors found that the attitudes of health advocates and policy-makers towards food and beverage companies are softer (or less determined) compared to the tobacco and alcohol industries.^12^ Over 25% of the survey respondents reported difficulty in deciding their stance on the food and beverage industries because food is necessary, unlike tobacco and alcohol, and the structure of the food industry is more diverse than alcohol; it comprises beneficial products and actors as well as harmful ones. Such ambiguity regarding the trustworthiness of food and beverage companies should be reconsidered; this paper’s analysis makes it clear that the food and beverage industries aggressively opposed SSB taxes in the consultative process of the 2018 HLM, despite clear evidence for its effectiveness in addressing NCDs.^33,50^

 The impact of including other industries in multi-stakeholder consultations should be further assessed. It is worth noting that there was no industry representing polluting industries in the negotiation process of the 2018 HLM. This probably lowered the political cost to the inclusion of environmental risk factors in the Declaration. Future multi-stakeholder governance should seriously consider the risk of including any commercial entities that have conflicts of interest. As others have already argued in 2011, “legitimate engagement with industry does not require that corporations be given such a prominent seat at the policy-making table.”^14^

 Furthermore, we need to think of the management of industry interference as a task that requires more than merely barring certain industries from multi-stakeholder consultations. Even if certain industries were to be excluded from interactive hearings, they would still attempt to influence global policy-making by lobbying Member States in-country or as invited parties to some Member State delegations to political processes. In a way, documented multi-stakeholder consultations make industry positions and activities more visible, such that stricter criteria for participation might encourage even greater lobbying ‘behind closed doors.’ Excluding certain industries from consultation processes on NCDs is more likely to facilitate robust political commitments if complemented with comprehensive safeguards against industry interference at international, national and local levels.

## Conclusion

 In order to better understand the inadequate global response to NCDs, this paper assessed the policy positions of different stakeholders in relation to the 2018 Political Declaration on NCDs. Through a frame analysis of 159 publicly available documents submitted during the negotiation process of the Declaration, a typology of policy positions was developed. A comparison of draft and final versions of the Declaration revealed not only differences in the policy positions of different stakeholders, but differences in their political power and capacity to influence the final text of the Declaration, as reflected in the changes between the draft and final versions. It should be widely acknowledged that the current form of multi-stakeholder governance, which includes commercial entities such as alcohol, food and beverage companies as ‘stakeholders,’ poses a serious risk to progress in the global response to NCDs.

## Acknowledgements

 We thank Suvi Huikuri, Juana Cooke, and Elfatih Abdelraheem for useful feedback.

## Ethical issues

 The ethical issue is not applicable since this paper is based on the policy documents and statements that are publicly available on the WHO and the UN websites.

## Competing interests

 Authors declare that they have no competing interests.

## Authors’ contributions

 MS wrote and revised the paper and contributed to data analysis and interpretation. DW led the conceptual design of the study, contributed to the collection of data, and provided comments on drafts. RS contributed to clarification of writing and provided comments on drafts. All authors read and approved the final manuscript.
